# Handling Poor Accrual in Pediatric Trials: A Simulation Study Using a Bayesian Approach

**DOI:** 10.3390/ijerph18042095

**Published:** 2021-02-21

**Authors:** Danila Azzolina, Giulia Lorenzoni, Silvia Bressan, Liviana Da Dalt, Ileana Baldi, Dario Gregori

**Affiliations:** 1 Unit of Biostatistics, Epidemiology and Public Health, Department of Cardiac Thoracic Vascular Sciences and Public Health, University of Padova, 35128 Padova, Italy; danila.azzolina@uniupo.it (D.A.); giulia.lorenzoni@unipd.it (G.L.); ileana.baldi@unipd.it (I.B.); 2 Department of Translational Medicine, University of Eastern Piedmont, 28100 Novara, Italy; 3 Department of Women’s and Children’s Health, University of Padova, 35128 Padova, Italy; silvia.bressan.1@unipd.it (S.B.); liviana.dadalt@unipd.it (L.D.D.)

**Keywords:** power-prior, poor accrual, Bayesian trial

## Abstract

In the conduction of trials, a common situation is related to potential difficulties in recruiting the planned sample size as provided by the study design. A Bayesian analysis of such trials might provide a framework to combine prior evidence with current evidence, and it is an accepted approach by regulatory agencies. However, especially for small trials, the Bayesian inference may be severely conditioned by the prior choices. The Renal Scarring Urinary Infection (RESCUE) trial, a pediatric trial that was a candidate for early termination due to underrecruitment, served as a motivating example to investigate the effects of the prior choices on small trial inference. The trial outcomes were simulated by assuming 50 scenarios combining different sample sizes and true absolute risk reduction (ARR). The simulated data were analyzed via the Bayesian approach using 0%, 50%, and 100% discounting factors on the beta power prior. An informative inference (0% discounting) on small samples could generate data-insensitive results. Instead, the 50% discounting factor ensured that the probability of confirming the trial outcome was higher than 80%, but only for an ARR higher than 0.17. A suitable option to maintain data relevant to the trial inference is to define a discounting factor based on the prior parameters. Nevertheless, a sensitivity analysis of the prior choices is highly recommended.

## 1. Introduction

Difficulties in the enrolment of the overall trial sample size, as indicated at the design stage, could be caused by several factors (i.e., high costs, regulatory barriers, narrow eligibility criteria, and cultural attitudes toward research in almost all research fields). Effects can be different depending on the population’s characteristics and the intervention under evaluation [1].

Prior research evaluating the reasons for termination across a broad range of trials reported that insufficient enrolment is the most common reason, with a frequency ranging from 33.7% to 57%, depending on the definition used [2,3]. The slow or low accrual problem is common in clinical research on adults, primarily in oncology [4,5,6] and cardiology [7], as well as in pediatric research, in which 37% of clinical trials are terminated early due to inadequate accrual [8]. Pediatrics is a research field that requires particular attention, since accrual issues are associated with methodological and ethical challenges [9]. It is essential to consider that the management and conduct of pediatric trials are more complicated than those of adult trials in terms of practical, ethical, and methodological problems [10].

From a statistical point of view, low accrual results in a reduced sample size, compromising the ability to accurately answer the primary research question due to a reduction in the likelihood of detecting a treatment effect [11]. The scientific community has conveyed that early termination of a trial due to poor accrual leads to inefficiency in clinical research, with consequent increases in costs [12] and a waste of resources, as well as a waste of the efforts of the children involved in the trial [13].

For these reasons, alternative and innovative approaches to pediatric clinical trial design have been a recent topic of debate in the scientific community [9,14]. Alternative methods for pediatric trial design and analysis have been proposed by recent guidelines in the field, i.e., the ICH (International Council for Harmonisation) Topic E11 guidelines [15], the guidance for trial planning and design in the pediatric context [16], and the EMA (European Medicines Agency) guidelines [16,17,18].

It is noteworthy that data from trials terminated prematurely for poor accrual can provide useful information for reducing the uncertainty about the treatment effect in a Bayesian framework [11]. 

In recent years, Bayesian methods have increasingly been used in the design, monitoring, and analysis of clinical trials due to their flexibility [19,20]. Considering the research setting described in this work, the Bayesian methods used for accrual monitoring are also interesting [21]. These methods are well suited to designing and analyzing studies conducted with small sample sizes and are particularly appropriate for studies involving children, even in cases of rare disease outcomes [9]. 

In clinical trials that are candidates for early termination due to poor accrual reasons, a Bayesian approach may be useful for incorporating the available knowledge on the investigated treatment effect, reported in the literature or elicited by experts’ opinions [22]. In addition, in a Bayesian setting, prior information combined with data may support the final inference for a trial conducted on a limited number of enrolled patients [23,24]. 

In pediatric trials, for example, the awareness that a treatment is effective in adults increases the probability of its efficacy in children. This knowledge may be quantitatively translated into a prior probability distribution [9,14].

However, when there is a small sample size, the final inference may be severely conditioned by a misleading prior definition [24]. In this framework, the Food and Drug Administration (FDA) suggests performing a sensitivity analysis on prior definitions [25], especially for very small sample sizes [26]. In this regard, the power prior approach is used to design and analyze small trials to control for the weight of historical information, translated into prior distributions, through prior discounting factors [27,28]. The use of historical information to define the prior distribution in a nonparametric context is a method recently used in the literature [29]. Informative prior elicitation is typically a challenging task even in the presence of historical data (objective prior) [30]. Ibrahim and Chen [28] proposed the power prior approach to incorporate the historical data in the analysis of a current study. The method is based on the raising of the likelihood function of the historical data to a power parameter between 0 and 1 (power parameter). This parameter represents the proportion of the historical data incorporated in the prior. 

Hobbs modified the conventional approach, accounting for commensurability of the information in the historical and current data to determine how much the historical information is used in the inference [31]. Other power-prior proposals calibrate the type I error by controlling the degree of similarity between the new and historical data [32,33]. The prior-data conflict has also been addressed and incorporated in the power prior in a commensurability parameter defined by using a measure of distribution distance in a group sequential design clinical trial [34]. A mixture of priors, for the one-parameter exponential family, has been also considered in a sequential trial, to incorporate the historical data accounting for rapid reaction to prior-data conflicts by adding an extra weakly-informative mixture component [35].

In general, the power prior approach is widely used for the design and analysis of clinical trial data. The method is useful for handling problems related to a lack of exchangeability between the historical and current data, and the risk that prior information overwhelms the clinical trial data information [27].

The optimal amount of discounting factors for an informative prior remains to be discussed [14].

This study investigated the effects of the prior choices on the final inference, especially for studies conducted with limited sample sizes, such as pediatric trials. A pediatric trial candidate for early termination due to underrecruitment, the RESCUE trial, served as a motivating example for the simulation study proposed.

A set of possible trial outcomes were simulated. The simulation plan was designed to evaluate the effects of the prior choices on the trial results by evaluating different scenarios depending on the number of patients involved in the study and the magnitude of the true treatment effect.

## 2. Materials and Methods

### 2.1. Motivating Example

The RESCUE trial was a randomized controlled double-blind trial. The purpose of the study was to evaluate the effect of adjunctive oral steroids in preventing renal scarring in young children and infants with febrile urinary tract infections. The primary outcome was the renal scar absolute risk reduction (ARR) between the treatment arms. The study was designed expecting an ARR of 0.20 to determine a renal scar reduction from 40% to 20%.

After two years, only 17 recruited patients completed the follow-up for the study outcome (6 in treatment and 11 in control) due to procedural problems and poor compliance with the study therapy and final diagnosis [16,17,18]. 

### 2.2. Simulation Plan

The possible trial outcomes were simulated by assuming several scenarios combining different sample sizes and true ARRs. The simulated data were analyzed via the Bayesian approach using a beta prior distribution whose parameters were derived from trials conducted in research settings similar to the RESCUE trial. The beta-binomial model was considered because it is the most widely used approach among the Bayesian methods to summarize event rates in clinical trials [36]. This parametrization is easily computationally tractable and is very precise [37].

Informative, low-informative, and uninformative priors were selected for the analyses according to the discounting levels placed on the prior parameters.

The classical, non-Bayesian approach was considered a benchmark. 

This simulation study is defined by:

1. Data generation hypotheses.
2. Analysis of simulated data.
3. Presentation of the results of simulations.

A flowchart synthesizing the simulation plan is reported in Figure S1, Supplementary Material.

### 2.3. Data Generation Hypotheses

#### 2.3.1. Simulation Scenarios

The simulation plan consisted of 50 scenarios. Each scenario represents a single combination of the treatment effect (ARR) and the sample size used to generate the data. Fifty scenarios were considered, since they combined ten different sample sizes (ranging from 15 to 240) within five assumed ARRs (Table 1). The ARR ranged from −0.07 to −0.27, with an increment of 0.07, according to the treatment effects suggested by the literature [38,39].

#### 2.3.2. Data Generation within Scenarios

For each scenario, the trial data were randomly generated 5000 times. The data were drawn from a binomial random variable, assuming a true event rate in the control arm of $\pi_{\mathrm{control}\;}=0.33$. This event rate is in-between the results provided by Huang et al. [38] and Shaikh et al. [39] for the control group.

The treatment arm data were generated using a binomial random variable hypothesizing an ARR, one for each experiment, in compliance with the simulation plan provided in Table 1, where the sample size is showed overall. However, it is assumed that the control arm contains 60% of the sample size to reflect the group imbalance in the motivating example.

### 2.4. Analysis of the Simulated Data

The 5000 randomly generated data points were analyzed via the Bayesian method by considering: (1) the informative prior, (2) the low-informative prior, and (3) the uninformative prior. A frequentist analysis was performed for comparison purposes. 

The data were simulated 5000 times by a binomial random variable in a frequentist approach. For each of the repeated simulations, the ARR was calculated and the binomial confidence interval was estimated.

#### 2.4.1. Prior Definition

A mixture of beta priors was considered for the outcome evaluation, using data provided by the literature [38,39]. The clinical trial results were combined in a mixture of distributions. The beta distributions comprising the mixture of priors for each scar event rate in the treatment and control groups were derived from other trials’ historical information [27].

The functional form of the distribution is characterized by the shape α and scale β parameters Π∼Beta(α,β) [40], where Π is the parameter that characterizes the event rate on which to make inference. The shape value α is defined by the number of events x observed in other trials, while the β value corresponds to the number of subjects not experiencing the event (n−x) [41].

1. Huang et al. [38] reported probabilities of scarring of ${\hat{\pi }}_{\mathrm{treat}\;\left(\mathrm{Huang}\right)}=\frac{6}{18}=0.33$ and ${\hat{\pi }}_{\mathrm{control}\;\left(\mathrm{Huang}\right)}=\frac{39}{65}=0.66$ in the treatment and control arms, respectively. Considering this information, the informative beta prior can be derived as:
  $$\Pi_{\mathrm{treat}\;\left(\mathrm{Huang}\right)}∼\mathrm{Beta}\left(6,12\right)$$
  $$\Pi_{\mathrm{control}\;\left(\mathrm{Huang}\right)}∼\mathrm{Beta}\left(39,26\right)$$
2. Shaikh et al. [39] reported, instead, probabilities of scarring of ${\hat{\pi }}_{\mathrm{treat}\;\left(\mathrm{Shaikh}\right)}=0.098$ (12|123) and ${\hat{\pi }}_{\mathrm{control}\;\left(\mathrm{Shaikh}\right)}=0.168$ (22|131) in the treatment and control arms, respectively. Considering this information, the informative beta prior can be derived as:
  $$\Pi_{\mathrm{treat}\;\left(\mathrm{Shaikh}\right)}∼\mathrm{Beta}\left(12,111\right)$$
  $$\Pi_{\mathrm{control}\left(\mathrm{Shaikh}\right)}∼\mathrm{Beta}\left(22,109\right)$$

The information was combined in a mixture of beta priors:

- For the treatment arm, the beta mixture is defined as:
  $$\Pi_{\mathrm{treat}\;}={\gamma \Pi }_{\mathrm{treat}\;\left(\mathrm{Huang}\right)}+\left(1-\gamma \right)\Pi_{\mathrm{treat}\;\left(\mathrm{Shaikh}\right)}$$
  The expected value for the mixture random variable is, for the treatment arm, a weighted mean of the expectations over the mixture components:
  $$E\left[\Pi_{\mathrm{treat}\;}]\;=\gamma E[\Pi_{\mathrm{treat}\left(\mathrm{Huang}\right)}\right]+\left(1-\gamma \right)E[\Pi_{\mathrm{treat}\left(\mathrm{Shaikh}\right)}]$$
  If we denote the beta shape $\alpha_{\mathrm{treat}\left(\mathrm{Huang}\right)}$ and $\alpha_{\mathrm{treat}\left(\mathrm{Shaikh}\right)}$, respectively for the Huang and Shaikh studies, and $\beta_{\mathrm{treat}\left(\mathrm{Huang}\right)}$ and $\beta_{\mathrm{treat}\left(\mathrm{Shaikh}\right)}$ the scales for the considered studies, the mixture expected value may be computed as:
  $$E\left[\Pi_{\mathrm{treat}\;}\right]=\gamma E\left[\Pi_{\mathrm{treat}\left(\mathrm{Huang}\right)}\right]+\left(1-\gamma \right)E\left[\Pi_{\mathrm{treat}\left(\mathrm{Shaikh}\right)}\right]$$
  $$E\left[\Pi_{\mathrm{treat}\;}\right]=\gamma \frac{\alpha_{\mathrm{treat}\left(\mathrm{Huang}\right)}}{\alpha_{\mathrm{treat}\left(\mathrm{Huang}\right)+}\beta_{\mathrm{treat}\left(\mathrm{Huang}\right)}}+\left(1-\gamma \right)\frac{\alpha_{\mathrm{treat}\left(\mathrm{Shaikh}\right)}}{\alpha_{\mathrm{treat}\left(\mathrm{Shaikh}\right)+}\beta_{\mathrm{treat}\left(\mathrm{Shaikh}\right)}}$$
  $$=\gamma \frac{6}{6+12}+\left(1-\gamma \right)\frac{12}{12+111}$$
  If we assume an equal weight value γ=0.5, $E\left[\Pi_{\mathrm{treat}\;}\right]=0.215$.
- The mixture variance is given by:
  $$\begin{array}{c} \mathrm{Var}\left[\Pi_{\mathrm{treat}\;}\right]=\left[\gamma \left(\mathrm{Var}\left[\Pi_{\mathrm{treat}\left(\mathrm{Huang}\right)}\right]+E\left[\Pi_{\mathrm{treat}\left(\mathrm{Huang}\right)}\right]-E\left[\Pi_{\mathrm{treat}\;}\right]\right)\right]+\\ +\left[\left(1-\gamma \right)\left(\mathrm{Var}\left[\Pi_{\mathrm{treat}\left(\mathrm{Shaikh}\right)}\right]+E\left[\Pi_{\mathrm{treat}\left(\mathrm{Shaikh}\right)}\right]-E\left[\Pi_{\mathrm{treat}\;}\right]\right)\right] \end{array}$$
  where the variances of the mixture components are:
  $$\mathrm{Var}\left[\Pi_{\mathrm{treat}\left(\mathrm{Huang}\right)}\right]=\frac{\alpha_{\mathrm{treat}\left(\mathrm{Huang}\right)}\beta_{\mathrm{treat}\left(\mathrm{Huang}\right)}}{{\left(\alpha_{\mathrm{treat}\left(\mathrm{Huang}\right)+}\beta_{\mathrm{treat}\left(\mathrm{Huang}\right)}\right)}^{2}(\alpha_{\mathrm{treat}\left(\mathrm{Huang}\right)+}\beta_{\mathrm{treat}\left(\mathrm{Huang}\right)}+1)}$$
  $$\mathrm{Var}[\Pi_{\mathrm{treat}\left(\mathrm{Shaikh}\right)}]=\frac{\alpha_{\mathrm{treat}\left(\mathrm{Shaikh}\right)}\beta_{\mathrm{treat}\left(\mathrm{Shaikh}\right)}}{{\left(\alpha_{\mathrm{treat}\left(\mathrm{Shaikh}\right)+}\beta_{\mathrm{treat}\left(\mathrm{Shaikh}\right)}\right)}^{2}(\alpha_{\mathrm{treat}\left(\mathrm{Huang}\right)+}\beta_{\mathrm{treat}\left(\mathrm{Shaikh}\right)}+1)}$$
  Equal weight was assumed for the components of the mixture, therefore, γ=0.5, $E\left[\Pi_{\mathrm{treat}\;}\right]=0.215$, and SD[$\Pi_{\mathrm{treat}\;}]\;$= 0.08.
- For the treatment arm, the mixture is defined as:
  $$\Pi_{\mathrm{control}\;}={\gamma \Pi }_{\mathrm{control}\;\left(\mathrm{Huang}\right)}+\left(1-\gamma \right)\Pi_{\mathrm{control}\;\left(\mathrm{Huang}\right)}$$
  $$\mathrm{with}\;E\left[\Pi_{\mathrm{control}\;}\right]=0.38\;\mathrm{and}\;\mathrm{SD}[\Pi_{\mathrm{control}\;}]\;=0.05\;\mathrm{and}\;\gamma =0.5.$$

#### 2.4.2. Discounting the Priors: The Power Prior Approach

Different levels of penalization (discounting) were provided for the historical information using a power prior approach [28] to perform a sensitivity analysis on the prior choices. The historical information can be included in the final inference using a $\mathrm{Beta}\left(\alpha_{1},\beta_{1}\right)$ prior, where:

$$\alpha_{1}=1+\alpha_{0}d_{0}$$

$$\beta_{1}=1+\beta_{0}d_{0}$$

The $\alpha_{0}$ ${\mathrm{and}\;\beta }_{0}$ values are the parameters defined by the number of successes and failures derived from the literature and are $\alpha_{0}$ and $\beta_{0}$, respectively. The value $d_{0}$ defines the amount of historical information to be included in the final inference. The discounting factor is otherwise defined as $\left(1-d_{0}\right)\times 100$ and represents the level of penalization (discounting) of the historical information derived from other studies.

1. If $d_{0}$ = 0, the data provided by the literature are not considered, indicating a 100% discount of the historical information. According to this scenario, the prior is an uninformative Beta(1,1) distribution.
2. If $d_{0}\;$= 1, then all of the information provided by the literature is considered in setting the prior, indicating a 0% discount of the historical data.

Analyses of the simulated trials were conducted using three different priors:

- Power prior without discounting (informative, $d_{0}$ = 1). A beta informative prior was derived considering the number of successes and failures found in the literature [42], as defined in the method section.
- Power prior 50% discounting (low-informative, $d_{0}$= 0.5). The beta prior with a 50% discount, defined in the literature as a substantial-moderate discounting factor [43], was defined based on the beta parameters comprising the mixture of priors specified in the informative scenario.
- Power prior 100% discounting (uninformative, $d_{0}$= 0). A mixture of Beta(1,1) priors was defined.

##### Effective Sample Size (ESS) Calculation

The ESS was computed on the mixture of beta distribution by using the Morita approach to quantify the prior influence on the final inference using the RBesT package in R (R Foundation for Statistical Computing, Vienna, Austria) [44]. For the mixture of beta prior (equal weight) without power prior discounting ($d_{0}\;$*=* 1), an ESS of 55 and 98 was achieved for treatment and control arm. However, discounting the beta parameters for $d_{0\;}$*=* 0.5 (low-informative prior), the ESS is equal to 24 and 48. 

**Figure 1:**
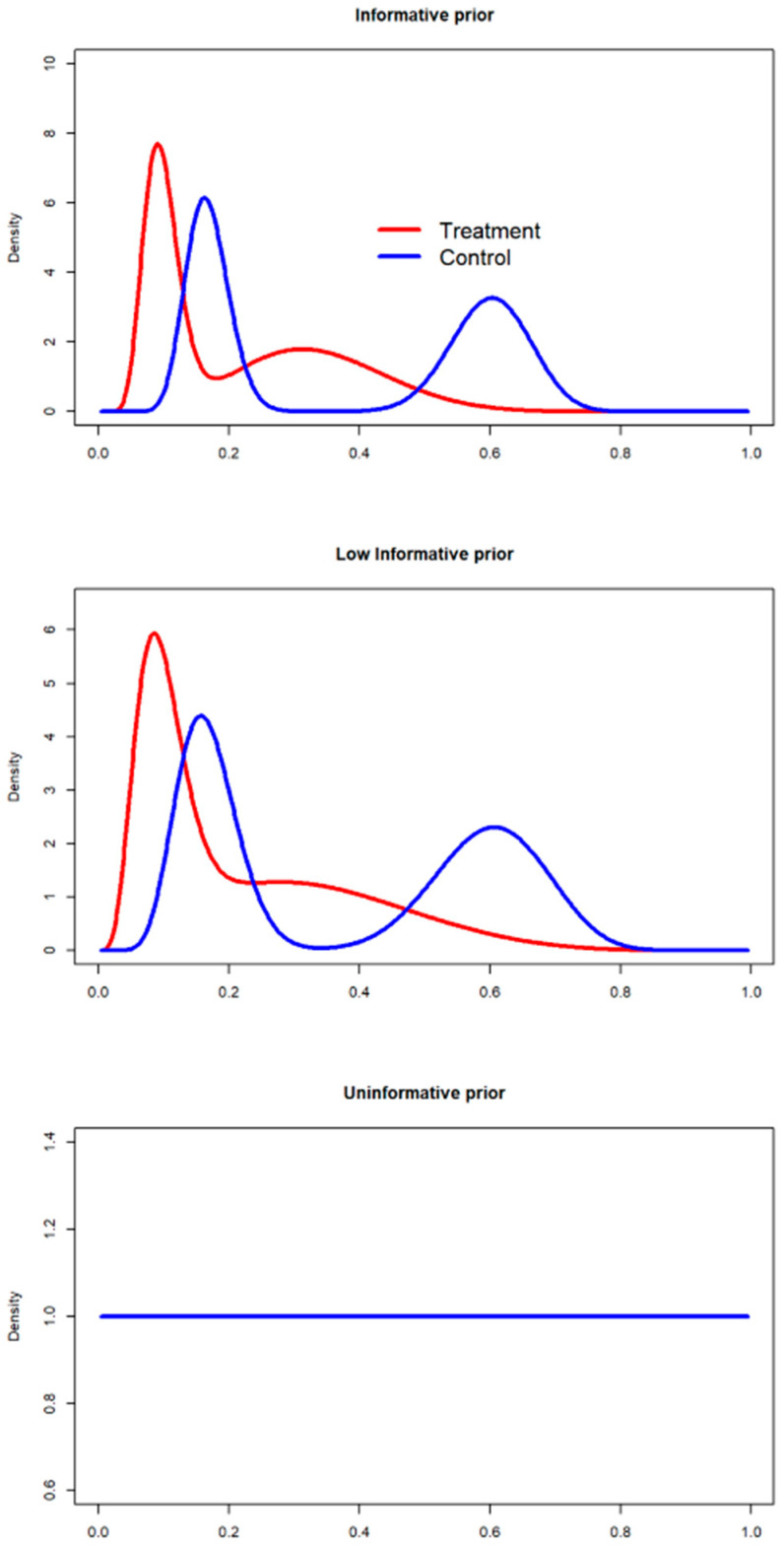
Prior distributions: The prior distributions are defined by an equal-weighted mixture (γ=0.5) of beta priors. The components of the mixture prior are, for the treatment arm, $\Pi_{\mathrm{treat}\;\left(\mathrm{Huang}\right)}∼\mathrm{Beta}\left(6,12\right)$ and $\Pi_{\mathrm{treat}\;\left(\mathrm{Shaikh}\right)}∼\mathrm{Beta}\left(12,111\right)$. The mixture of priors (γ=0.5 ) for the control arm is defined by $\Pi_{\mathrm{control}\;\left(\mathrm{Huang}\right)}∼\mathrm{Beta}\left(39,26\right)$ and $\Pi_{\mathrm{control}\left(\mathrm{Shaikh}\right)}∼\mathrm{Beta}\left(22,109\right)$. No discounting on the beta priors parameters has been provided ($d_{0}$ = 1) for the Informative priors. The information has been partially discounted for the low-informative prior scenario ($d_{0}$ = 0.5). The priors parameters are full discounted for the uninformative prior scenario ($d_{0}$ = 0), collapsing to a Beta(1,1) distribution.

The prior distributions are presented in Figure 1.

#### 2.4.3. Posterior Estimation

A beta-binomial model was employed to analyze the difference in event rates between arms [45]. The posterior distribution for the ARR outcome requires the estimation of the posterior distribution of the scar proportion in each arm separately, and was computed with the following Markov chain Monte Carlo (MCMC) resampling procedure [46]:

- A first resampling of the proportion of scarring $\Pi_{\mathrm{treat}}^{*}$ from $\Pi_{\mathrm{treat}}|X_{\mathrm{treat}}$, which is the posterior distribution for the treatment group.
- A second resampling of $\Pi_{\mathrm{control}}^{*}$ from $\Pi_{\mathrm{control}}|X_{2}$.
- The posterior distribution for the parameter related to the difference in proportions was obtained by calculating $\mathrm{ARR}=\Pi_{\mathrm{treat}}^{*}-\Pi_{\mathrm{control}}^{*}$ from the distributions previously resampled [47].

The resampling procedures were performed using an MCMC estimation algorithm, as indicated in the literature [46], using 3 chains, 6000 iterations, and 1000 adaptations. 

An example of the inference results is reported in the Supplementary Material, showing the priors and the posterior distributions calculated on a single database generated by assuming an ARR equal to 0.17.

The computations were performed using OpenBUGS (Free Software Foundation, Boston, MA, USA) [48] and R version 3.3.2 [49]; the simulation R codes are reported in the Supplementary Material.

#### 2.4.4. Convergence Assessment

The Geweke method [50] was considered to assess the convergence of the MCMC results within iterations. Geweke’s statistics test was computed for each analysis conducted on the simulated data. Geweke’s Z-score plot was also visually inspected to assess the convergence.

### 2.5. Results of the Simulations

Four sets of 200 results summarizing 50 scenarios in combination with four methods of analysis were defined as:

1. The proportion of the 5000 simulated trials for which the credibility intervals (CIs), or confidence intervals, for a frequentist analysis do not contain an ARR equal to 0. The proportion of intervals not containing the 0 and containing the data generator ARR was also calculated.
2. The mean length across 5000 simulated trials of the CI.
3. The mean of the posterior median estimate across 5000 simulated trials or the mean of the point-estimated ARR across 5000 simulated trials for the frequentist analysis.
4. The mean absolute percentage error (MAPE):
  $$\mathrm{MAPE}=\frac{1}{n}\sum_{t=1}^{n}\left|\frac{{\mathrm{ARR}}_{\mathrm{true}}-{\hat{\mathrm{ARR}}}_{t}}{{\mathrm{ARR}}_{\mathrm{true}}}\right|$$
  ${\mathrm{ARR}}_{\mathrm{true}}$ is the true treatment effect considered to generate the data; ${\hat{\mathrm{ARR}}}_{t}$ is the estimated treatment effect (posterior median, or point estimate, for the frequentist analysis) achieved for each simulation t within the n = 5000 simulated trials.

## 3. Results

**Figure 2:**
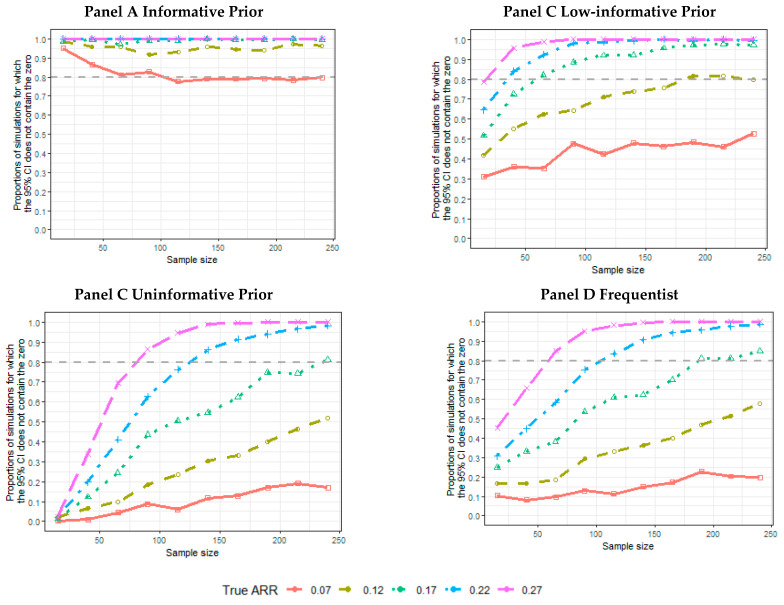
The proportion of CIs within simulated trials not including the zero absolute risk reduction (ARR) according to the sample size, and true ARR for informative prior (Panel A), low-informative prior (Panel B), uninformative prior (Panel C), and frequentist analysis (Panel D).

The proportion of 5000 simulated trials ensuring that the 95% CI does not contain an ARR equal to zero is greater than 90% for all of the informative scenarios, even if the sample size is smaller than 50, except for the 0.07 true ARR. For the 0.07 ARR, this proportion declines as the data used to estimate the likelihood increases (Figure 2, Panel A). This proportion is higher than 80% only for sample sizes greater than 70, and the true ARR is greater than 0.17 for the low-informative priors (Figure 2, Panel B). The pattern of the simulation results is similar, considering the proportion of simulations for which the CI does not include the 0, and includes the true data generator ARR (Figure S3, Supplementary Material).

Similar behavior is observed among the uninformative Bayesian (Figure 2, Panel C) and frequentist (Figure 2, Panel D) estimates, for which this proportion reaches 80% for an ARR greater than 0.22 and sample sizes greater than 120.

**Figure 3:**
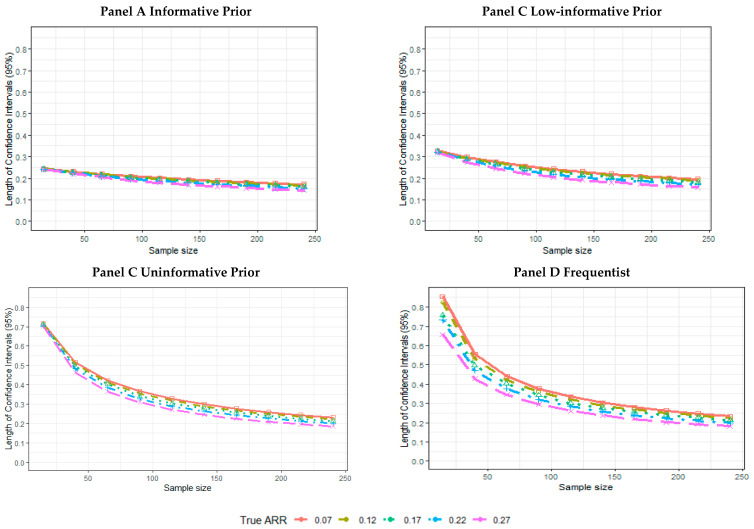
Simulation results for the 95% CI length according to the sample size and true ARR for informative prior (Panel A), low-informative prior (Panel B), uninformative prior (Panel C), and frequentist analyses (Panel D).

The 95% CI length decreases as the sample size increases for all of the Bayesian parametrizations and the frequentist estimates (Table S1, Supplementary Material). The informative (Figure 3, Panel A) and low-informative priors (Figure 3, Panel B) showed more variability in the posterior length of the CIs across different true ARR values. The CI lengths are more similar for different data generation ARR assumptions for the uninformative (Figure 3, Panel C) and frequentist (Figure 3, Panel D) simulations. In general, especially for smaller sample sizes, the estimates are less precise for the frequentist and Bayesian uninformative prior scenarios than for the informative and low-informative prior estimates (Table 1).

**Figure 4:**
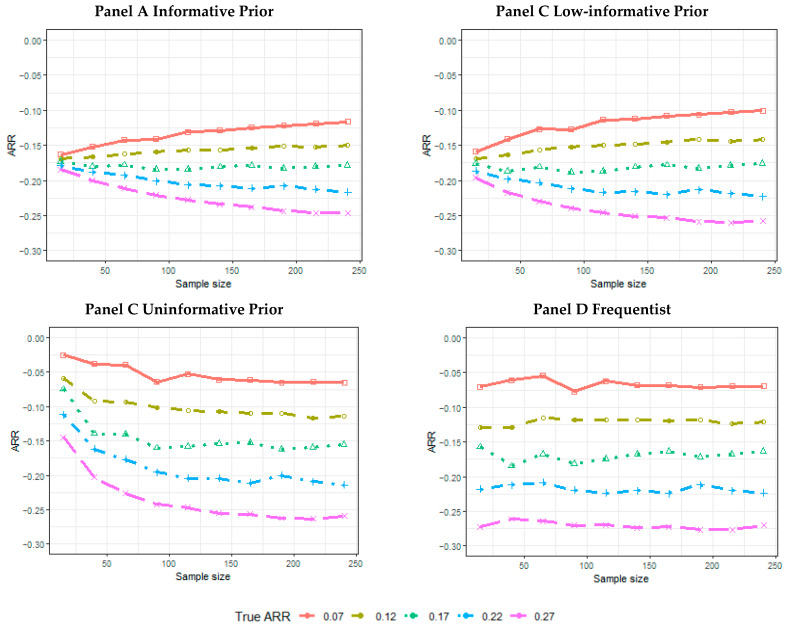
Simulation results for the estimated ARR (posterior median, or point estimate, for frequentist analysis) according to the sample size and true ARR for informative prior (Panel A), low-informative prior (Panel B), uninformative prior (Panel C), and frequentist analyses (Panel D).

The posterior median ARR estimates are influenced by the prior choices, especially for the informative prior. The estimated ARRs are similar to each other for smaller sample sizes across the true treatment effect, while the posterior median ARR estimates converge to the true ARR for larger sample sizes (Figure 4, Panel A). A similar pattern is observed for the low-informative scenarios; however, for smaller sample sizes, greater variability in the posterior median estimates is observed across the different ARRs used to generate the data (Figure 4, Panel B). The ARR is overestimated for small sample sizes in the uninformative prior scenarios (Figure 4, Panel C). Instead, the frequentist estimates across the simulated trial are similar to the true treatment effect for all of the sample sizes (Figure 4, Panel D).

The MAPE estimate decreases as the sample size increases for all the prior parametrizations (Table S1, Supplementary Material). A lower true ARR (i.e., 0.07) ensures a decreasing effect that is more evident than a higher true ARR (Table S1, Supplementary Material).

**Figure 5:**
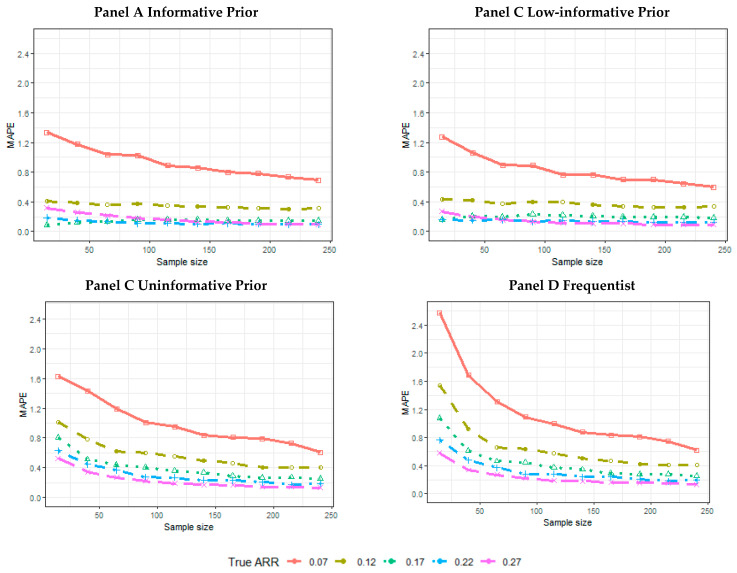
Simulation results for the mean absolute percentage error (MAPE) estimate according to the sample size and true ARR for informative prior (Panel A), low-informative prior (Panel B), uninformative prior (Panel C), and frequentist analyses (Panel D).

Also, the MAPE seems to be constant for a higher true ARR in informative (Figure 5, Panel A) and low-informative prior (Figure 5, Panel B) simulations. For the uninformative (Figure 5, Panel C) and frequentist scenarios (Figure 5, Panel D), instead, a reduction in MAPE is also evident for higher true ARR values. The MAPE values are higher for the frequentist scenarios than all of the Bayesian estimates, including those provided via the uninformative prior (Table S1, Supplementary Material).

The hypothesis of the stationarity of the chain was not rejected according to Geweke’s statistic for all of the analyses conducted on the simulated data and for all of the prior parametrizations. The Z-scores within iterations was also visually inspected. An example within simulations (ARR = 0.07 and sample size = 65) is reported in the Figure S2, Supplementary Material. The Z-score lies within the acceptance stationarity region (±2) or all chains and all the prior parametrizations; the pattern is very similar for all the considered scenarios.

An example of a possible inference result is shown in the Supplementary Material. The posteriors were calculated for a generated trial data reporting 8 events over 56 in the treatment arm (${\hat{\pi }}_{\mathrm{treat}\;}$= 0.14) and 30 events over 84 in the control arm (${\hat{\pi }}_{\mathrm{control}\;}$= 0.36). The data generator ARR is 0.17, while the observed ARR is 0.22. Considering the different priors, the inference results are located in mean on the same event rate; however, the uncertainty in the posterior distribution increases, considering the uninformative prior assumption (Figure S4, Supplementary Material).

## 4. Discussion

Regulatory agencies advocate an increase in pediatric research, which is motivated by the need for more information on treatment labeling to guide pediatricians and to offer more suitable and safe treatments for children [14]. However, in various cases, pediatric trials have demonstrated difficulties in enrolling participants [51]. The RESCUE trial represents a typical example of a complex trial in pediatric research affected by poor accrual. The difficulties encountered in the enrolment and retention of participants are related to procedural problems related to the study protocol [51,52] and poor adherence to the therapy.

Bayesian data analysis may overcome challenges in the conduction of trials similar to the RESCUE study, allowing investigators to combine information provided by current trial data with evidence provided by the literature, as recommended by regulatory agencies to deal with small sample sizes [15].

The present findings show that Bayesian inference can detect a small treatment effect for small sample sizes (lower than 50), even if the prior is fully uninformative compared to a maximum likelihood approach. This result confirms the potential benefits of using a Bayesian method on small sample sizes. However, the literature suggests paying attention to the use of uninformative prior distributions for small clinical trials, because there is the possibility of including in the final inference extreme treatment effects that are potentially unexpected from a clinical point of view. For this reason, it is suggested to use evidence from previous trials to inform these prior distributions [53].

For this reason, a key issue in Bayesian analysis is the choice of prior. This simulation study demonstrated that, especially for small studies, the trial results could be influenced by the prior choices and weakly influenced by the data when using fully informative priors. In particular, a prior distribution incorporating favorable treatment effect information on small sample sizes is likely to conditionate the inference in favor of the treatment, even if, in truth, the effect is null or minimal. All of this implies that the prior in these contexts should be defined by using validated empirical evidence [27]. Conversely, this study suggests that the full informative prior elicited by considering large effect size tends to direct the inference towards the existence of a treatment effect for all the sample size scenarios. For this reason, we recommend, especially when the treatment effect hypothesized for the study design is large and the sample size is small, the use of a low-informative prior for achieving more data-driven results.

The situation is different if a discounting factor is placed on the prior parameters. Looking at the estimated values of ESS, the historical information retained in the prior in the low-informative scenario is halved, compared to the informative parameterization. This implies that the inference is more data-oriented, assuming a discounting of 0.5. The probability of confirming the trial results is demonstrated to be more data-dependent and, for sample sizes less than 50, is higher than 80% only for ARRs higher than 0.17.

As the power prior parameter increases, the prior becomes more informative, and the estimated precision (length of CI) increases. Looking at the differences between observed and estimated ARR, the inferential results, comparing the various parameterizations of the prior discounting factor, tend to converge toward the same conclusions in the direction of the generating data effect size starting from a sample size of 150 subjects. All this implies that, for studies conducted on a considerable number of patients, it is possible to tune the prior toward a more informative solution ($d_{0}$ > 0.5), obtaining results representing a suitable compromise between the available historical information and what is suggested by the data.

In the literature, some reasons are addressed for a suitable discounting of historical prior information. First, the historical data and the current trial evidence may be heterogeneous concerning the study design and conduct [28]. Moreover, as also demonstrated by this simulation analysis, especially for small trials, an informative historical prior may overwhelm the current trial evidence [27].

Another issue outlined in this paper is the potentially misleading information on the treatment effect provided by the posterior median effect for a sample size smaller than 50 patients. This source of bias is evident not only for informative inference but also for low-informative and uninformative analyses. Conversely, the frequentist point estimate is unbiased in terms of the mean because of the proportion estimator’s asymptotical unbiasedness over repeated resamples. However, in the frequentist approach, the variability of results across sample replications is very high for small samples, even though the effect, on average, is unbiased [54]. Bayesian estimates, on the other hand, return scenarios of inferential results that are less variable, especially if a minimum amount of historical information is incorporated into the prior.

The frequentist approach considers all the parameters to be fixed; the data are a realization of a random variable. Instead, Bayesian methods assume that all the parameters are random and the data are fixed [54]. This point of view leads to incorporating the available knowledge on the prior parameters into a probability distribution. For this reason, it is important to ensure that the information on which informative priors are based is accurate; otherwise, the resulting estimates and posterior standard deviations could be biased if misleading informative priors are utilized [55].

In this regard, the Bayesian approach leads to thinking about inference in terms of a probability distribution on the treatment effect, rather than a point estimate or confidence interval. Therefore, a Bayesian approach is oriented toward a progressive uncertainty reduction (on a posterior probability distribution) in treatment effect estimation. Historical information contributes sequentially to the reduction of this uncertainty [56]. The uncertainty can be measured in terms of the CI width. The simulation results demonstrate a narrower CI for small sample sizes (similarly across different true ARRs) for Bayesian analyses compared to the frequentist approach. This effect has also been reported in the literature [57].

The present results show that Bayesian methods can outperform frequentist methods with small samples by providing increased efficiency and an increased ability to determine non-null effects. However, the appropriate prior distribution choice, especially on small datasets, plays a fundamental role. Researchers might need to consult experts, meta-analyses, or review studies in the area of interest to obtain informative, accurate priors that can meaningfully contribute to posterior distributions. Furthermore, a sensitivity analysis on priors (i.e., defining the robustness of conclusions that may be affected by decisions made on the priors) is highly recommended for pediatric trials [14], which is in line with the literature [24] and FDA recommendations [25].

### Study Limitations

This study was conducted considering only the conjugate prior beta setting. It may be interesting to explore the impact of inference in the posterior case obtained in a nonclosed form. For example, instead of directly placing a parameter derived on the beta prior, it may be advisable to consider expert elicitation about treatment effects to define the specific prior distribution. Moreover, future research development is needed to investigate the effect of an eventual prior-data conflict on the trial results according to different study size.

## 5. Conclusions

Bayesian inference is a flexible tool compared to frequentist inference, especially for trials conducted in a poor accrual setting. A full informative Bayesian inference, conducted on small samples, can generate data-insensitive results. On the other hand, the use of an uninformative prior distribution may include, in the final inference, clinically unproven extreme treatment effect hypotheses. A power prior approach on sample sizes smaller than 50 patients seems to be a good compromise between these two methods. However, the choice of parameters and discounting factors should be negotiated with expert pediatricians and should be guided by an appropriate consultation of the scientific literature. In agreement with the FDA recommendations, a sensitivity analysis of priors is highly recommended.

## Figures and Tables

**Table 1:** Simulation scenarios.

**Scenario**	**1**	**2**	**3**	**4**	**5**	**6**	**7**	**8**	**9**	**10**	**11**	**12**	**13**	**14**	**15**	**16**	**17**	**18**	**19**	**20**	**21**	**22**	**23**	**24**	**25**
**Sample size**	15	40	65	90	115	140	165	190	215	240	15	40	65	90	115	140	165	190	215	240	15	40	65	90	115
**True ARR**	0.07	0.07	0.07	0.07	0.07	0.07	0.07	0.07	0.07	0.07	0.12	0.12	0.12	0.12	0.12	0.12	0.12	0.12	0.12	0.12	0.17	0.17	0.17	0.17	0.17
**Scenario**	**26**	**27**	**28**	**29**	**30**	**31**	**32**	**33**	**34**	**35**	**36**	**37**	**38**	**39**	**40**	**41**	**42**	**43**	**44**	**45**	**46**	**47**	**48**	**49**	**50**
**Sample size**	140	165	190	215	240	15	40	65	90	115	140	165	190	215	240	15	40	65	90	115	140	165	190	215	240
**True ARR**	0.17	0.17	0.17	0.17	0.17	0.22	0.22	0.22	0.22	0.22	0.22	0.22	0.22	0.22	0.22	0.27	0.27	0.27	0.27	0.27	0.27	0.27	0.27	0.27	0.27

ARR=Absolute Risk Reduction.

## Data Availability

Data sharing not applicable.

## Supplementary Materials

The following are available online at https://www.mdpi.com/1660-4601/18/4/2095/s1, Figure S1: Simulation Plan, Figure S2: Geweke’s Z-statistics for Informative, Figure S3: The proportion of CIs within simulated trials, Figure S4. Prior and posterior density estimates, Table S1: Simulation results according to the prior choices, and Simulation Codes.
