# Supplementary material for: Handling Poor Accrual in Pediatric Trials: A Simulation Study Using a Bayesian Approach
Source: Int J Environ Res Public Health. 2021 Feb 21;18(4):2095. doi: 10.3390/ijerph18042095 (PMC7924849; doi:10.3390/ijerph18042095)
Supplement: Supplementary file 1 [file ijerph-18-02095-s001.pdf]

Supplementary Material

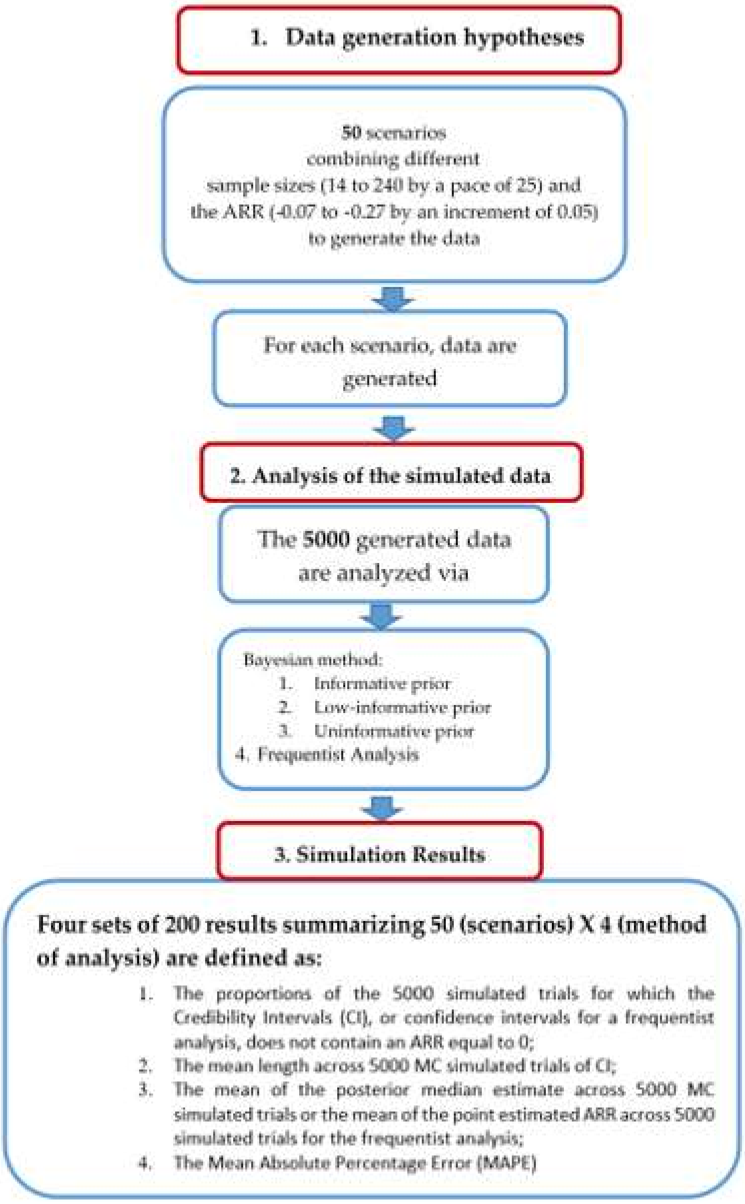

Figure S1 Simulation Plan

Table S1. Simulation results according to the prior choices. The percentage of simulations not including the zero ARR, the CI length, the posterior median of the ARR, and the MAPE estimates are reported for each simulation scenario corresponding to a combination of the true ARR used to generate the data and the sample size.

		Informative				Low-Informative				Uninformative				Frequentist			
**Sample Size**	**True ARR.**	**% CI not including zero**	**Length**	**posterior median**	**MAPE**	**% CI not including zero**	**Length**	**posterior median**	**MAPE**	**% CI not including zero**	**Length**	**posterior median**	**MAPE**	**% CI not including zero**	**Length**	**posterior median**	**MAPE**
15	-0.07	0.950	0.245	-0.163	1.335	0.310	0.327	-0.159	1.274	0.003	0.717	-0.025	1.624	0.103	0.854	-0.070	2.574
40	-0.07	0.867	0.231	-0.152	1.177	0.360	0.298	-0.141	1.061	0.010	0.515	-0.038	1.430	0.080	0.555	-0.061	1.688
65	-0.07	0.810	0.220	-0.142	1.037	0.353	0.276	-0.127	0.893	0.043	0.422	-0.040	1.194	0.097	0.441	-0.055	1.311
90	-0.07	0.827	0.210	-0.141	1.024	0.477	0.257	-0.127	0.889	0.087	0.367	-0.064	1.010	0.130	0.377	-0.077	1.092
115	-0.07	0.777	0.202	-0.131	0.889	0.423	0.243	-0.114	0.764	0.060	0.329	-0.053	0.949	0.113	0.335	-0.062	0.997
140	-0.07	0.790	0.194	-0.129	0.857	0.480	0.231	-0.112	0.764	0.117	0.300	-0.061	0.839	0.150	0.304	-0.069	0.877
165	-0.07	0.790	0.187	-0.125	0.801	0.463	0.220	-0.108	0.697	0.130	0.277	-0.062	0.811	0.173	0.281	-0.069	0.837
190	-0.07	0.793	0.182	-0.122	0.781	0.483	0.210	-0.106	0.696	0.170	0.259	-0.065	0.789	0.227	0.262	-0.071	0.812
215	-0.07	0.783	0.176	-0.119	0.735	0.460	0.202	-0.103	0.645	0.190	0.244	-0.064	0.731	0.203	0.246	-0.070	0.747
240	-0.07	0.797	0.171	-0.116	0.694	0.527	0.195	-0.101	0.596	0.170	0.231	-0.065	0.611	0.197	0.233	-0.070	0.620
15	-0.12	0.987	0.244	-0.169	0.410	0.420	0.325	-0.169	0.430	0.020	0.715	-0.059	1.012	0.167	0.822	-0.129	1.536
40	-0.12	0.957	0.230	-0.166	0.391	0.553	0.294	-0.164	0.417	0.067	0.507	-0.093	0.779	0.167	0.533	-0.129	0.921
65	-0.12	0.960	0.218	-0.162	0.363	0.623	0.271	-0.156	0.375	0.097	0.413	-0.094	0.622	0.187	0.423	-0.116	0.665
90	-0.12	0.917	0.207	-0.160	0.369	0.643	0.252	-0.153	0.404	0.183	0.357	-0.102	0.596	0.293	0.361	-0.118	0.635
115	-0.12	0.930	0.198	-0.157	0.355	0.713	0.237	-0.149	0.394	0.237	0.318	-0.106	0.550	0.330	0.321	-0.118	0.577
140	-0.12	0.957	0.190	-0.156	0.339	0.737	0.224	-0.148	0.365	0.303	0.288	-0.107	0.493	0.363	0.289	-0.118	0.507
165	-0.12	0.947	0.184	-0.153	0.323	0.757	0.214	-0.145	0.343	0.330	0.268	-0.110	0.464	0.400	0.269	-0.119	0.473
190	-0.12	0.940	0.178	-0.151	0.318	0.817	0.205	-0.142	0.327	0.400	0.250	-0.110	0.408	0.467	0.251	-0.118	0.419
215	-0.12	0.973	0.172	-0.152	0.308	0.817	0.197	-0.144	0.326	0.463	0.236	-0.117	0.404	0.513	0.237	-0.124	0.415
240	-0.12	0.963	0.167	-0.149	0.314	0.797	0.189	-0.141	0.336	0.520	0.223	-0.114	0.401	0.577	0.223	-0.121	0.413
15	-0.17	0.987	0.243	-0.173	0.091	0.517	0.323	-0.176	0.165	0.013	0.709	-0.075	0.808	0.250	0.756	-0.157	1.077
40	-0.17	0.993	0.226	-0.180	0.128	0.727	0.288	-0.186	0.211	0.123	0.494	-0.140	0.515	0.333	0.498	-0.185	0.608
65	-0.17	0.973	0.214	-0.178	0.131	0.820	0.265	-0.181	0.199	0.243	0.403	-0.140	0.443	0.383	0.405	-0.167	0.463

90	-0.17	0.990	0.202	-0.184	0.158	0.887	0.244	-0.188	0.230	0.437	0.343	-0.160	0.407	0.537	0.342	-0.181	0.442
115	-0.17	0.990	0.193	-0.184	0.156	0.920	0.228	-0.186	0.216	0.507	0.303	-0.158	0.357	0.610	0.301	-0.174	0.373
140	-0.17	1.000	0.185	-0.181	0.156	0.923	0.217	-0.181	0.210	0.547	0.278	-0.154	0.337	0.623	0.277	-0.167	0.345
165	-0.17	0.993	0.179	-0.179	0.147	0.957	0.206	-0.178	0.192	0.623	0.257	-0.152	0.292	0.703	0.256	-0.163	0.295
190	-0.17	0.997	0.172	-0.182	0.151	0.973	0.196	-0.182	0.192	0.747	0.239	-0.162	0.270	0.813	0.239	-0.171	0.276
215	-0.17	1.000	0.167	-0.180	0.151	0.977	0.189	-0.179	0.192	0.743	0.226	-0.159	0.273	0.813	0.225	-0.168	0.275
240	-0.17	0.997	0.161	-0.178	0.145	0.973	0.181	-0.176	0.180	0.813	0.213	-0.155	0.257	0.850	0.212	-0.163	0.256
15	-0.22	1.000	0.242	-0.178	0.189	0.647	0.322	-0.186	0.161	0.020	0.708	-0.112	0.633	0.307	0.732	-0.218	0.761
40	-0.22	1.000	0.224	-0.188	0.149	0.840	0.283	-0.198	0.150	0.197	0.483	-0.162	0.445	0.450	0.472	-0.212	0.476
65	-0.22	1.000	0.210	-0.193	0.139	0.923	0.257	-0.203	0.158	0.410	0.386	-0.177	0.370	0.583	0.377	-0.209	0.369
90	-0.22	1.000	0.197	-0.201	0.112	0.983	0.235	-0.212	0.131	0.627	0.328	-0.195	0.279	0.753	0.321	-0.219	0.284
115	-0.22	1.000	0.187	-0.206	0.112	0.987	0.218	-0.216	0.147	0.763	0.290	-0.204	0.272	0.837	0.284	-0.224	0.282
140	-0.22	1.000	0.179	-0.208	0.106	0.993	0.205	-0.216	0.136	0.860	0.263	-0.204	0.238	0.907	0.259	-0.220	0.242
165	-0.22	1.000	0.171	-0.211	0.108	1.000	0.195	-0.220	0.141	0.913	0.243	-0.211	0.234	0.943	0.239	-0.225	0.238
190	-0.22	1.000	0.165	-0.208	0.104	0.997	0.186	-0.214	0.127	0.940	0.226	-0.200	0.209	0.957	0.223	-0.212	0.203
215	-0.22	1.000	0.159	-0.213	0.094	1.000	0.178	-0.219	0.119	0.967	0.212	-0.209	0.176	0.977	0.210	-0.220	0.179
240	-0.22	1.000	0.153	-0.217	0.099	0.997	0.169	-0.223	0.129	0.983	0.199	-0.214	0.185	0.987	0.197	-0.223	0.190
15	-0.27	1.000	0.241	-0.184	0.318	0.787	0.319	-0.196	0.273	0.023	0.702	-0.145	0.530	0.453	0.659	-0.273	0.581
40	-0.27	1.000	0.221	-0.200	0.259	0.957	0.275	-0.218	0.194	0.340	0.463	-0.203	0.345	0.657	0.428	-0.261	0.338
65	-0.27	1.000	0.205	-0.211	0.218	0.987	0.245	-0.230	0.158	0.693	0.365	-0.226	0.269	0.850	0.344	-0.264	0.270
90	-0.27	1.000	0.191	-0.221	0.183	1.000	0.223	-0.239	0.133	0.863	0.310	-0.242	0.226	0.953	0.296	-0.270	0.222
115	-0.27	1.000	0.179	-0.228	0.156	1.000	0.205	-0.245	0.114	0.947	0.271	-0.247	0.191	0.983	0.260	-0.269	0.188
140	-0.27	1.000	0.170	-0.234	0.136	1.000	0.192	-0.251	0.108	0.990	0.246	-0.255	0.176	0.997	0.237	-0.274	0.178
165	-0.27	1.000	0.162	-0.238	0.125	1.000	0.182	-0.253	0.107	0.997	0.226	-0.256	0.168	1.000	0.218	-0.272	0.165
190	-0.27	1.000	0.154	-0.244	0.105	1.000	0.171	-0.258	0.093	1.000	0.208	-0.262	0.148	1.000	0.202	-0.276	0.154
215	-0.27	1.000	0.149	-0.246	0.100	1.000	0.164	-0.260	0.094	1.000	0.196	-0.264	0.143	1.000	0.191	-0.276	0.148
240	-0.27	1.000	0.144	-0.246	0.100	1.000	0.158	-0.258	0.094	1.000	0.186	-0.259	0.135	1.000	0.181	-0.271	0.135

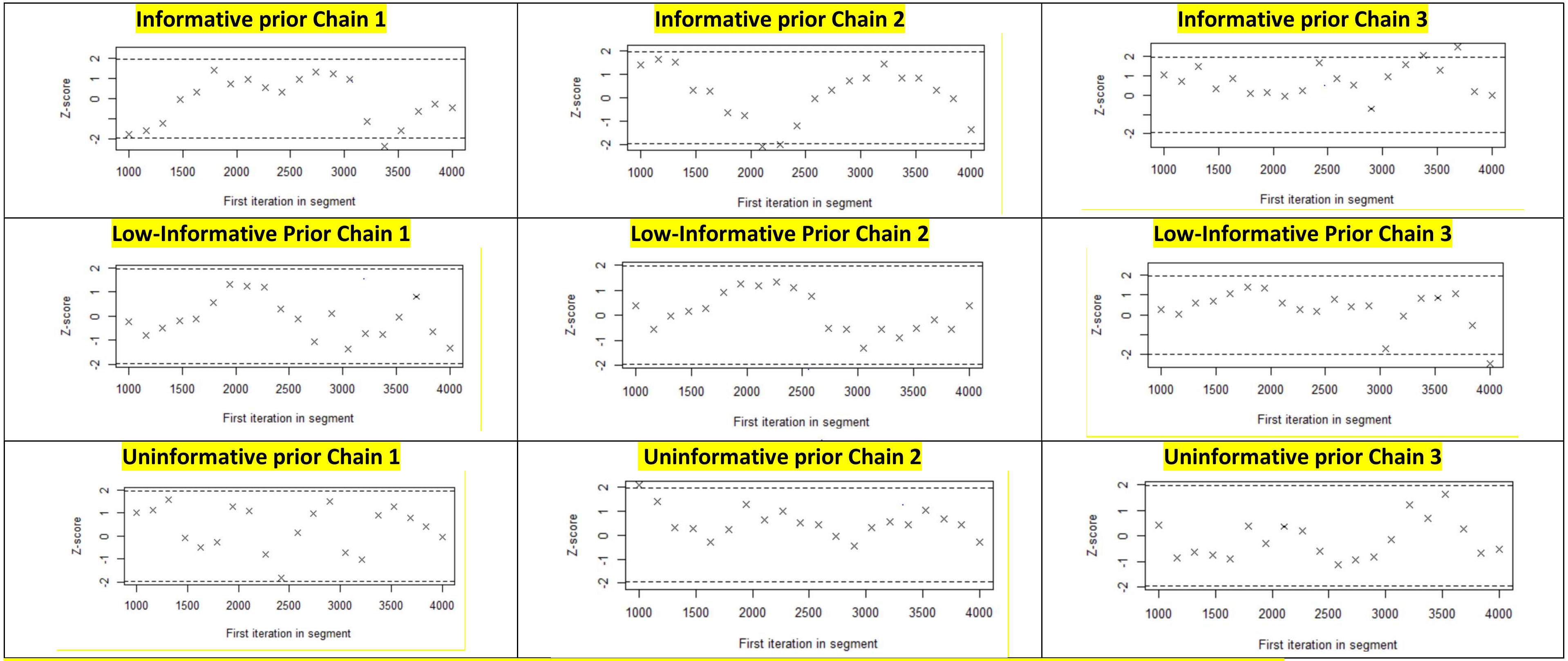

Figure S2 Geweke's Z-statistics for Informative, Low informative, and Uninformative priors, ARR=0.07 and sample size=65

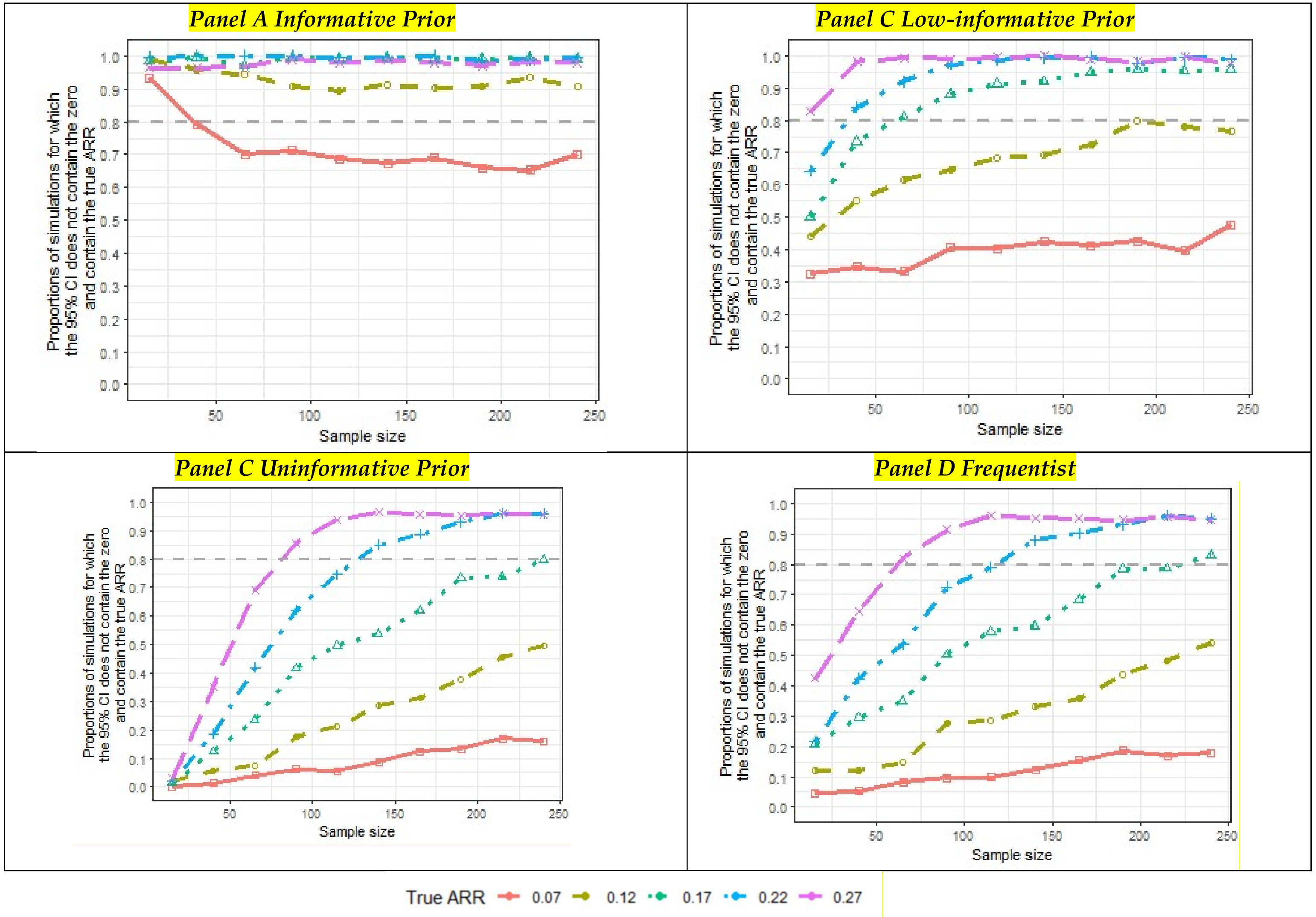

Figure S3. The proportion of CIs within simulated trials not including the zero and including the true ARR according to the sample size and true ARR for informative prior (Panel A), low-informative prior (Panel B), uninformative prior (Panel C), and frequentist analysis (Panel D).

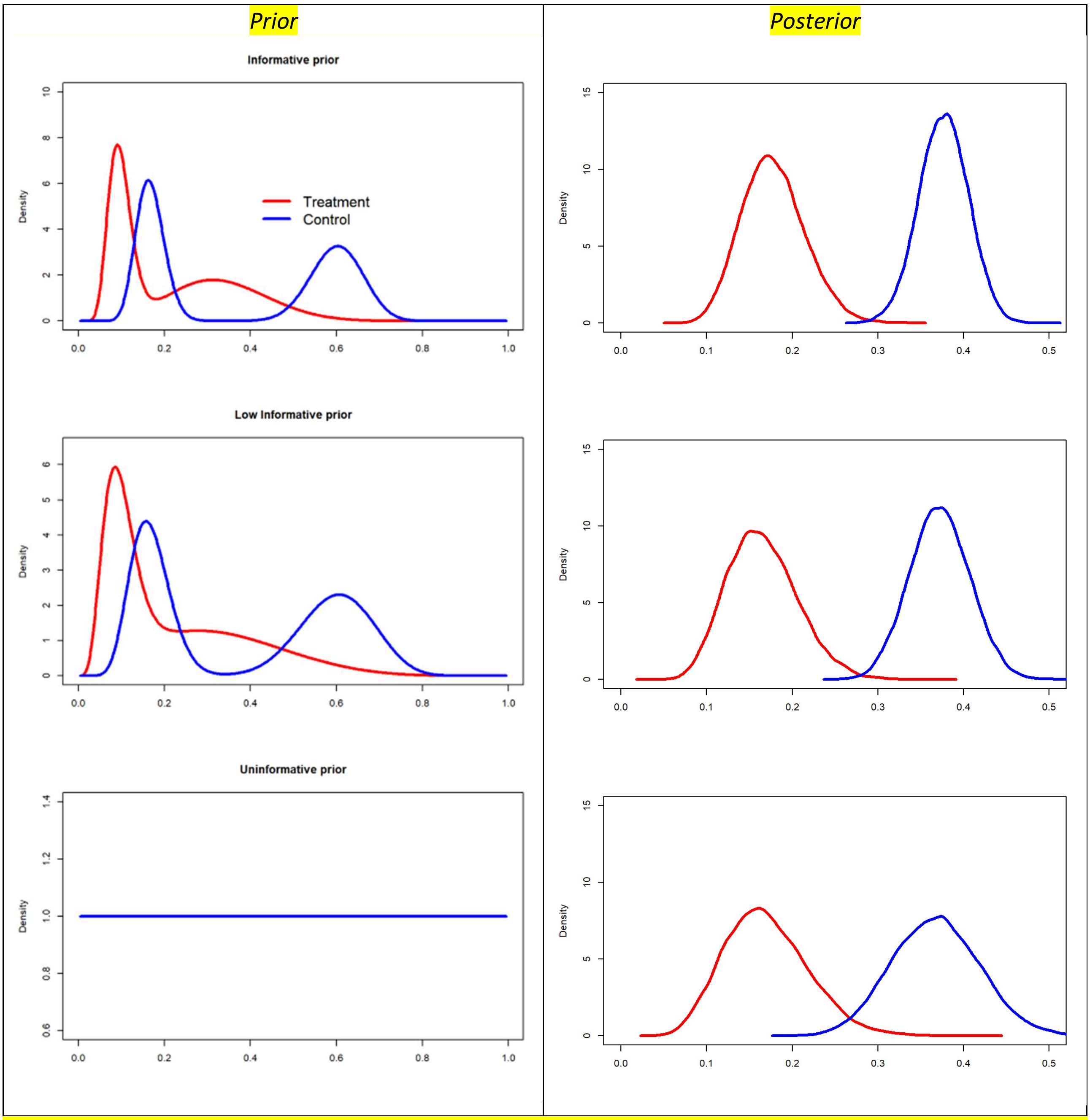

Figure S4. Prior and posterior density estimates. The posteriors have been calculated for a generated trial data reporting 8 events over 56 in the treatment arm ($\hat{\pi}_{treat}$ = 0.14) and 30 events over 84 in the control arm ($\hat{\pi}_{control}$ = 0.36). The data generator ARR is 0.17; the observed ARR is 0.22.

Simulation Codes

```
library(rjags)


# sample sizes
ss<-seq(15,255,25)

# True ARR
red<-seq(0.07,0.27,0.05)

# allocation between samples
ratio<-0.6

#true event rate in control arm
p1pa<-0.33

#scenarios
scen<-expand.grid(ss,red,ratio)
colnames(scen)<-c("ss","red","ratio")


# simulate data function
simulate_data<-function(x){

####sample sizes
n1<-(scen$ss[x])*(1-scen$ratio[x])
n2<-((scen$ss[x])*(scen$ratio[x]))

#number of simulated trial
nrep<-5000

# store posterior median, prop of CI excluding 0, length and convergence assessment
q5.mu.theta<-c()
re<-c()
len<-c()
cond<-c()

for (i in 1:nrep) {

#simulate from a binomial in treatment reducing by ARR
exp<-rbinom(p=p1pa-scen$red[x],n1,1)
a<-sum(exp)
ra<-a/n1

#simulate from a binomial in control
ctr<-rbinom(p=p1pa,n2,1)
b<-sum(ctr)
rb<-b/n2

#true and observed ARR
diffe_oss<-ra-rb
diffe_true<--scen$red[x]

###model

model_string <- "model {
s_1 ~ dbin(theta_1,n_1)
s_2 ~ dbin(theta_2,n_2)

#Informative priors
theta_1.1 ~ dbeta(1+6,1+12)
theta_1.2 ~ dbeta(1+12,1+123-12)


theta_2.1 ~ dbeta(1+39,1+26)
theta_2.2 ~ dbeta(1+22,1+131-22)

#mixture
```

```
theta_1<-0.5*theta_1.1+0.5*theta_1.2
theta_2<-0.5*theta_2.1+0.5*theta_2.2


diff <- theta_1-theta_2
P <-( step(diff - (0)))
}"

###Jags model and samples
model <- jags.model(textConnection(model_string), data =
list(s_1=a,n_1=n1,s_2=b,n_2=n2),
                    n.chains = 3, n.adapt=1000,quiet=T)
samples <- coda.samples(model,  c("theta_1","theta_2", "diff", "P"),
n.iter=6000,quiet=T)

##assess if convergence is achieved
###over analysis performed on the simulated trial
cond[i]<-sum(pnorm(abs(c(geweke.diag(samples)[[1]])$z[2:4]),lower.tail = T)>0.05)==3&
sum(pnorm(abs(c(geweke.diag(samples)[[2]])$z[2:4]),lower.tail = T)>0.05)==3&
sum(pnorm(abs(c(geweke.diag(samples)[[3]])$z[2:4]),lower.tail = T)>0.05)==3


#### estractposterior mean and 95% credible interval
mu.theta.out <- cbind(samples[[1]][,2], samples[[2]][,2], samples[[3]][,2])
attributes(mu.theta.out) <- NULL

q25.mu.theta <- quantile(mu.theta.out, .025)
q975.mu.theta <- quantile(mu.theta.out, .975)
q5.mu.theta[i] <- quantile(mu.theta.out, .5)

#conditions
re[i]<-q975.mu.theta<0&q25.mu.theta<0 #ci not including 0
len[i]<-abs(q25.mu.theta-q975.mu.theta) #ci length
}


perc<-mean(re)### how many trial results does not onclude 0
len<- mean(len) ###average length
est<-mean(q5.mu.theta) #average posterior median
mape<-mean(abs((q5.mu.theta-diffe_true)/diffe_true)) #mape average
conv<-(mean(cond))## how many times convergence is achieved mean
resu<-c(perc,len,est,mape,conv)

}


#store vectors with NA
scen$perc<-NA
scen$len<-NA
scen$est<-NA
scen$mape<-NA
scen$conv<-NA


# loop the function over scenarios filling the vector results
for (x in 1:nrow(scen)) {
  rr=simulate_data(x)
  scen$perc[x]<-rr[1]
  scen$len[x]<-rr[2]
  scen$est[x]<-rr[3]
  scen$mape[x]<-rr[4]
  scen$conv[x]<-rr[5]

  cat(paste("°°°°°°°°°°°°°NEW ITER SS",x))
}



##################################################
#########################PRIORS COMPOSING THE MIXTURE
#LOW INFORMATIVE
theta_1.1 ~ dbeta(1+6*0.5,1+12*0.5
theta_1.2 ~ dbeta(1+12*0.5,1+(123-12)*0.5)
```

```
theta_2.1 ~ dbeta(1+39*0.5,1+26*0.5)
theta_2.2 ~ dbeta(1+22*0.5,1+(131-22)*0.5)

# UNINFORMATIVE
theta_1.1 ~ dbeta(1,1)
theta_1.2 ~ dbeta(1,1)

theta_2.1 ~ dbeta(1,1)
theta_2.2 ~ dbeta(1,1)


##################################################
#########################SIMULATE DATA FUNCTION FREQUENTIST
simulate_data_freq<-function(x){


  n1<-(scen$ss[x])*(1-scen$ratio[x])
  n2<-((scen$ss[x])*(scen$ratio[x]))
  nrep<-300

  q5.mu.theta<-c()
  re<-c()
  len<-c()

  for (i in 1:nrep) {


    exp<-rbinom(p=p1pa-scen$red[x],n1,1)
    a<-sum(exp)
    ra<-a/n1

    ctr<-rbinom(p=p1pa,n2,1)
    b<-sum(ctr)
    rb<-b/n2

    diffe_oss<-ra-rb
    diffe_true<--scen$red[x]

    s<-prop.test(x=c(a,b), n=c(n1,n2), correct=FALSE)


    q25.mu.theta <- s$conf.int[1]
    q975.mu.theta <- s$conf.int[2]
    q5.mu.theta[i] <- (s$estimate[1]-s$estimate[2])

    re[i]<-q975.mu.theta<0&q25.mu.theta<0
    len[i]<-abs(q25.mu.theta-q975.mu.theta)

  }
```
